# Change of direction asymmetry across different age categories in youth soccer

**DOI:** 10.7717/peerj.9486

**Published:** 2020-07-27

**Authors:** Athos Trecroci, Alessio Rossi, Thomas Dos’Santos, Damiano Formenti, Luca Cavaggioni, Stefano Longo, F. Marcello Iaia, Giampietro Alberti

**Affiliations:** 1 Department of Biomedical Sciences for Health, Università degli Studi di Milano, Milan, Italy; 2 Department of Computer Science, University of Pisa, Pisa, Italy; 3 Human Performance Laboratory, Directorate of Sport, Exercise, and Physiotherapy, University of Salford, Greater Manchester, United Kingdom; 4 Department of Biotechnology and Life Sciences (DBSV), University of Insubria, Varese, Italy

**Keywords:** Limb dominance, Sprint, Change of direction, Imbalance, Youth

## Abstract

**Background:**

In youth, the development of change of direction (COD) and sprint performance is a key component for successfully competing in soccer across age. During a COD, the presence of directional asymmetries may be detrimental due to the unpredictable nature of the sport. Therefore, the aims of the study were to investigate asymmetries in COD ability and to examine the differences in COD and sprint performance across age in young soccer players.

**Methods:**

Sixty-eight sub-elite soccer players of different age categories (U18, U17, U16, U15) were tested on a 10-m linear sprint test and 90°COD (5-m entry and exit) test in both directions. Asymmetric index (AI) of COD deficit was obtained for dominant (fastest) and non-dominant directions (slowest).

**Results:**

The results showed that U16 were more asymmetrical than U18, U17, and U15 from large to moderate effects. The sprint time improved linearly across age with U18 and U15 displaying the fastest and slowest 10-m sprint performance (*p* < 0.01), respectively. Moreover, COD ability measured by COD deficit did not change across age (*p* > 0.05).

**Conclusion:**

Given the results of this study, practitioners are encouraged to assess asymmetries between dominant and non-dominant directions rather than solely players’ COD ability in young soccer players.

## Introduction

Change of direction (COD) actions (e.g., side-steps, swerves, turns, crossover steps, and by-pass maneuvers) are essential types of movements on the soccer pitch giving a player the chance to effectively evade or mark an opponent, to create space for his or her teammates, and to score a goal (Hart et al., 2014; Rouissi et al., 2015; Trecroci et al., 2018b). Performing an effective COD requires high lower limbs strength to manage rapid decelerations (producing eccentric force) and subsequent accelerations (producing concentric force) within a short time and in multiple directions (Trecroci et al., 2018c). Therefore, it is recommended to train and monitor COD ability throughout the season for improving soccer players performance (Brughelli et al., 2008; Dos’Santos et al., 2019b; Trecroci et al., 2016).

In soccer, a number of COD tests have been proposed to monitor COD ability, such as 505 test, modified Illinois COD test, COD and acceleration test, and modified agility *T*-test (Cuthbert, Dos’Santos & Jones, 2019; Dos’Santos et al., 2019b; Hachana et al., 2014; Lockie et al., 2013; Loturco et al., 2019; Loturco et al., 2018; Sassi et al., 2009; Sporis et al., 2010). Most of them use the total time to complete the test as an actual measure of the players’ COD ability. However, total time might not be the most appropriate variable representing COD ability, being also biased by linear sprint capacity (Nimphius et al., 2017; Nimphius et al., 2016). Nimphius et al. (2016) reported that in the 505 COD test, which includes a single 180° COD task within two linear 5-m sprints, an individual spends ∼70% of the time sprinting during the test. Thus, potential changes in total time may be mainly explained by the players’ acceleration capacity rather than their COD ability. To overcome this issue, the COD deficit parameter has been introduced to provide a more valid and isolated measure of the COD ability, without being confounded and biased by linear sprint capacity (Nimphius et al., 2016). To calculate the COD deficit, the performance time in a given linear sprint test over a certain distance is deducted from the total running time of a COD test over an equivalent distance.

Soccer is a single-leg-dominant sport, which often exacerbates the use of a specific body side or a preferred path to change direction, especially in young players (Rouissi et al., 2016a; Rouissi et al., 2016b). The presence of such a bilateral difference in young players reflects the presence of an asymmetry that should be quantified from both performance and injury risk perspectives (Dos’Santos et al., 2019a; Hart et al., 2014). Indeed, an inability to move efficiently in one direction as opposed to another could impede a player’s movement and performance within the pitch. However, limited studies have investigated COD asymmetry in young soccer players. In the study of Rouissi et al. (2016a), it was observed that ∼52% of under 18 (U18) soccer players exhibited significantly lower total times in the inversed pattern (the first COD was to the right) of the modified Illinois COD test compared with the original (where the dominant leg is largely involved). Nevertheless, this result should be taken with caution because, as mentioned above, the sole use of total time to assess COD ability can be misleading. In another study (Rouissi et al., 2016b), under 17 (U17) elite soccer players presented significantly better COD performance (measured by COD deficit and COD total time variables) in 45°, 90°, 135°, and 180° turns using the dominant leg to side step (i.e., kicking leg) compared with the non-dominant leg. The authors concluded that leg dominance could affect COD ability among young soccer players. However, considering leg dominance (i.e., kicking limb) instead of preferred direction (e.g., turning toward left or right) may lead to misinterpretations of potential bilateral differences because athletes do not necessarily perform faster COD with the kicking limb (Dos’Santos et al., 2019a; Dos’Santos et al., 2017). Moreover, no comparison was made between the COD deficit and total time (Dos’Santos et al., 2019a; Dos’Santos et al., 2017).

The fact that COD deficit calculation does not include the acceleration phase would provide diverse quantification of asymmetry compared with COD total time. In this regard, Dos’Santos et al. (2019b) quantified the COD asymmetry in the 505 COD test by deficit and total time according to the preferred direction in youth netball players. Beside observing meaningful differences between preferred and non-preferred directions of both COD deficit and total time, the authors also found that asymmetry percentage was greater for COD deficit than COD total time, indicating a different sensitivity between tests in detecting asymmetry-related differences. Moreover, the authors reported that for COD deficit almost half of the subjects exhibited asymmetries higher than 10%, a general threshold beyond that an individual’s asymmetry is considered substantial (Dos’Santos et al., 2017; Trecroci et al., 2018b). Given the importance of monitoring asymmetries during COD tasks in youth soccer, it remains unknown how they differ across age categories. During maturation, young players potentially experience a phase of “motor awkwardness” (Rommers et al., 2019), which often results in alterations of motor control. Thus, it may be feasible that a youth individual’s ability to coordinate complex movements during COD tasks would be altered, possibly affecting both total time and COD deficit. Further investigations on this topic could provide practical insights to optimize the youth training development.

Therefore, the aim of the study was twofold: (i) to examine asymmetries in COD performance based on COD deficit in youth soccer players across different age categories; (ii) to examine the differences in COD and sprint performance between all categories.

## Methods

### Subjects

Sixty-eight soccer players divided by age category (U18, *n* = 17; U17, *n* = 17; U16, *n* = 17; U15, *n* = 17) were recruited from the same youth soccer academy and tested during an in-season training period. Players’ characteristics are presented in Table 1. All subjects and their parents/guardians provided a written informed consent for their adhesion to participate in the investigation. The study was approved by the Ethical Committee of the Università degli Studi di Milano, in accordance with the Helsinki’s declaration (Approval number: 32/16).

**Table 1:** Players’ characteristics separated by age category.

Age category	Age (years)	Height (m)	Body mass (kg)	Training program (number of sessions per week)	Age at PHV
U18	17.33 ± 0.44	1.77 ± 0.07	66.62 ± 7.31	4	14.32 ± 0.45
U17	16.17 ± 0.36	1.76 ± 0.08	65.54 ± 9.25	4	14.27 ± 0.43
U16	15.39 ± 0.12	1.75 ± 0.06	63.29 ± 5.9	3	13.38 ± 0.34
U15	14.42 ± 0.22	1.68 ± 0.06	59.61 ± 8.05	3	13.33 ± 0.30

**Notes.**

U18 = under 18; U17 = under 17; U16 = under 16; U15 = under 15; PHV = peak height velocity

### Procedures

In this cross-sectional study, 68 sub-elite soccer players of different age category from U15 to U18 performed a 10-m linear sprint test and a 90°COD test (5-m entry and exit) with both dominant and non-dominant sides. Asymmetries in the 90°COD test were investigated using total running time and COD deficit parameters across all age categories. The asymmetry index (AI) was also computed to examine imbalances between dominant and non-dominant COD performance (total time and COD deficits) for each age category.

All subjects were familiar with the physical tests involved in the study as they were commonly included in the testing batteries and subsequent monitoring procedures. The experimental assessment was conducted following the procedures previously described by Trecroci et al. (2018c) in terms of the period within a competitive season (from September to October), and environment (an outdoor artificial turf), time of the day (i.e., from 5 p.m. to 7 p.m.), weather conditions (no wind or rain). Prior to testing, height, sitting height and body mass of each subject were recorded using a stadiometer (SECA 213, Germany) and a portable scale (813, Germany) to the nearest of 1.0 cm and 0.1 kg, respectively (Trecroci et al., 2020; Trecroci et al., 2018c). The age at peak height velocity was obtained by the equation of Mirwald et al. (2002). The testing sessions were arranged to assess sprint performance and COD ability in a randomized order. At the beginning of the session, each subject underwent a 5-minute warm-up period in a standardized process as previously adopted by Trecroci et al. (2018b). Immediately before each test, subjects were also invited to re-warm-up with skipping movements and dynamic stretching. All individuals wore the same clothing and footwear throughout the testing session. All subjects recovered 10 min between each test to avoid fatigue-induced effects (Trecroci et al., 2020; Trecroci et al., 2018a; Trecroci et al., 2018b; Trecroci et al., 2018c). An electronic timing gates system (Microgate, Bolzano, Italia) was used to measure the sprint and COD performance time (Trecroci et al., 2020; Trecroci et al., 2018a; Trecroci et al., 2018b; Trecroci et al., 2018c).

### Sprint performance

From a standing position (two-point staggered stance), each subject started sprinting when ready over a 10-m distance. They performed three maximal 10-m efforts interspersed by 2 min of passive recovery. The slowest time was considered in the analysis.

### 90° Change of direction (COD) test

**Figure 1:**
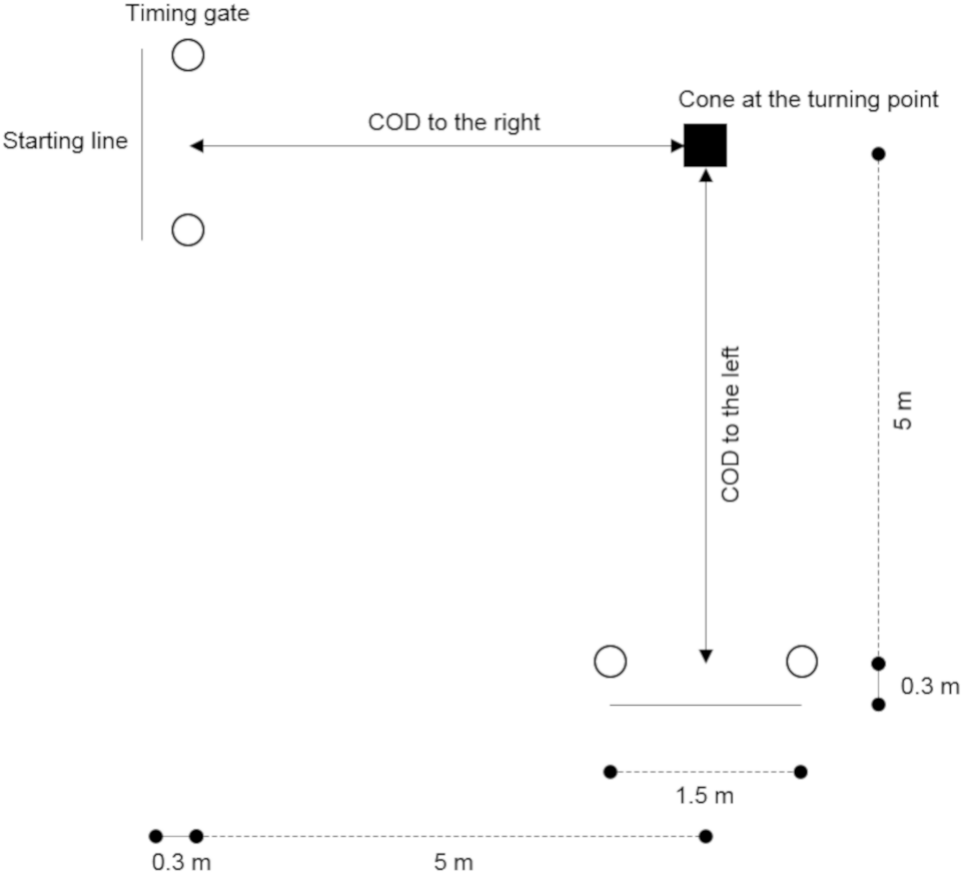
Layout of the 90° change of direction (COD) test.

Players were instructed to perform six sprints (three bouts for both right and left sides interspersed by 2 min of passive recovery) with a turning point at 90° represented by a cone (Fig. 1) (Trecroci et al., 2020). All participants were instructed to change direction at the turning point (around the cone) using the same side-step technique in each bout, to avoid any influence due to different COD execution technique (Rouissi et al., 2015). In case of hitting or touching the cone at the turning point, the player was stopped and invited to repeat the bout after 2 min of recovery. The 90°COD performance was measured by the total running time to complete the 5 m + 5 m course (COD_t_), and the fastest trial of each direction was used for further analysis. Furthermore, COD deficit (COD_d_) was calculated by subtracting the 10-m sprint time from the COD_t_ (Cuthbert, Dos’Santos & Jones, 2019; Nimphius et al., 2016; Trecroci et al., 2020).

### Asymmetric index calculation

Within the COD_t_ performance on right and left sides, the side with the lowest time was defined as the dominant direction (COD_t_-D), while the slowest was considered as the non-dominant direction (COD_t_-ND) (Dos’Santos et al., 2019b; Trecroci et al., 2020). Thus, the asymmetry index (AI%) of each player within a group was computed by the formula:

\begin{eqnarray*}\mathrm{AI}\text{%}=[({\mathrm{COD}}_{\mathrm{t}}\text{-}\mathrm{D}-{\mathrm{COD}}_{\mathrm{t}}\text{-}\mathrm{ND})/{\mathrm{COD}}_{\mathrm{t}}\text{-}\mathrm{D}]\times 100. \end{eqnarray*}

This calculation was also applied by using the same formula replacing the values with COD_d_ (Dos’Santos et al., 2019b). Moreover, as previously proposed by Dos’Santos et al. (2019b) and Trecroci et al. (2020) an asymmetry threshold (AT%) was also computed for each group by the following formula:

\begin{eqnarray*}\text{AT}\text{%}=\text{AI}\text{%}\text{mean}+(0.2\times \text{standard deviation of AI%mean}) \end{eqnarray*}

where AI%mean is the mean asymmetry index of each group. A player of an age group expressing an individual AI% value exceeding the corresponding AT% was classified as asymmetrical for that group.

### Statistical analysis

Data distribution was verified by conducting the Shapiro–Wilk’s test. Relative and absolute reliability were evaluated utilizing the Intra-class Correlation Coefficient (ICC), standard error of the measurement (SEM), and percent coefficient of variation (CV%), respectively. Paired *t*-tests were performed to investigate the difference between COD_t_-D and COD_t_-ND directions and COD_d_-D COD_d_-ND in each age category. The magnitude of each difference was detected by Cohen’s *d* effect size (*d*) with positive *d* indicating an effect favoring D over ND. A one-way analysis of variance (ANOVA) was used to evaluate potential differences across age categories (U18, U17, U16, and U15) for COD_t_-D and COD_t_-ND directions, and COD_d_-D COD_d_-ND directions as well as for 10-m sprint. In case of significance differences, a Tukey’s post-hoc test was used to control for multiple comparisons. Robust statistical method for non-normal data was also employed to detect between-group differences (e.g., AI% of U18 vs. AI% of U17) by effect size comparisons. The unscaled robust estimator for effect size *d* (*d*_r_) was calculated by the formula:

\begin{eqnarray*}{d}_{\mathrm{r}}=(Y1-Y2)/{s}_{\mathrm{p}}, \end{eqnarray*}

where Y1 and Y2 were the AI% trimmed means for the two categories to compare (e.g., U18 vs U17), and *s*_p_ was the square root of the pooled winsorized variance related to the same categories (Algina, Keselman & Penfield, 2005). To compute a trimmed mean and winsorized variance, a predetermined 10% of observations was removed at the ends of the distribution (Algina, Keselman & Penfield, 2005). The corresponding *d* and *d*_r_ were classified as *trivial* (< 0.2), *small* (0.2–0.5) *moderate* (0.5–0.8) and *large* (> 0.8). Ninety-five percent confidence interval (95% CI) for each effect size calculation was reported. Statistical analysis was performed using IBM Statistical Package for the Social Science version 21.0 (IBM Corp.; Armonk, NY, USA). The level of significance was set at an *α*-value of 0.05. Data are reported as mean  ±  SD.

## Results

ICC values showed excellent reliability in 10-m sprint (ICC = 0.95, 95% CI [0.93–0.97]), COD_t_-D (ICC = 0.94, 95% CI [0.89–0.96]), and COD_t_-ND (ICC = 0.95, 95% CI [0.83–0.98]). Absolute reliability was also obtained for 10-m sprint (SEM = 0.02 s, CV = 1.7%), COD_t_-D (SEM = 0.02 s, CV = 1.0%), and COD_t_-ND (SEM = 0.03 s, CV = 1%). Significant differences were observed in U18 (*d* = 0.57 and *d* = 0.83), U17 (*d* = 0.86 and *d* = 1.26), U16 (*d* = 1.13 and *d* = 1.44), and U15 (*d* = 0.70 and *d* = 0.63) between D and ND for both COD_t_ (*p* < 0.0001) and COD_d_ (*p* < 0.0001), respectively.

One-way ANOVA revealed significant differences across age category for 10-m sprint (*F*_(3,64)_ = 7.301, *p* < 0.001), COD_t_-D (*F*_(3,64)_ = 6.896, *p* < 0.001), COD_t_-ND (*F*_(3,64)_ = 5.524, *p* = 0.002), while non-significant differences were revealed for COD_d_-D (*F*_(3,64)_ = 1.814, *p* = 0.153) and COD_d_-ND (*F*_(3,64)_ = 1.294, *p* = 0.284). Post-hoc analysis for 10-m sprint revealed that U18, U17 and U16 were significantly faster than U15 (*p* < 0.001, *d* = 1.13 [1.88 to 0.38]; *p* = 0.038, *d* = 0.53 [−0.17 to 1.23]; *p* = 0.026, *d* = 0.74 [0.03 to 1.46], respectively). Post-hoc analysis for COD_t_-D revealed that U18 and U16 were significantly faster than U15 (*p* < 0.001, *d* = 1.15 [0.40 to 1.91]; *p* = 0.005, *d* = 1.04 [0.30 to 1.78], respectively). Likewise, post-hoc analysis for COD_t_-ND revealed that U18 were also faster than U15 (*p* = 0.001, *d* = 1.04 [0.30 to 1.78]) (Table 2).

**Table 2:** Performance outcomes of each variable and asymmetric indexes of COD_d_ for each age category. Ninety-five percent (95%) confidence intervals (CI) are shown in squared brackets.

Variables	**U18**	**U17**	**U16**	**U15**
**Sprint performance**				
10 m sprint (s)	1.75 ± 0.06^*^ [1.72 to 1.78]	1.79 ± 0.07 [1.75 to 1.82]	1.78 ± 0.05 [1.78 to 1.81]	1.83 ± 0.08 [1.81 to 1.88]
**COD ability**				
COD_t_-D (s)^+^	2.23 ± 0.07^*^ [2.18 to 2.26]	2.30 ± 0.08 [2.26 to 2.33]	2.24 ± 0.07^*^ [2.20 to 2.27]	2.33 ± 0.10 [2.28 to 2.39]
COD_t_-ND (s)	2.28 ± 0.10^*^ [2.22 to 2.32]	2.36 ± 0.09 [2.31 to 2.40]	2.31 ± 0.09 [2.28 to 2.36]	2.39 ± 0.11 [2.34 to 2.45]
COD_d_-D (s)^+^	0.47 ± 0.06 [0.44 to 0.50]	0.51 ± 0.06 [0.47 to 0.53]	0.45 ± 0.05 [0.42 to 0.48]	0.50 ± 0.09 [0.44 to 0.53]
COD_d_-ND (s)	0.52 ± 0.07 [0.48 to 0.55]	0.57 ± 0.07 [0.53 to 0.60]	0.53 ± 0.06 [0.50 to 0.56]	0.56 ± 0.10 [0.50 to 0.60]
**Asymmetric index (%)**				
COD_d_	−11.01 ± 12.38^a^^,^^c^^,^^d^ [−17.37 to −4.64]	−12.71 ± 9.71^b^ [−17.70 to −7.72]	−18.43 ± 12.11^e^ [−24.66 to 12.20]	−14.18 ± 16.00 [−22.41 to −5.95]

**Notes.**

COD_t_ change of direction total time COD_d_ change of direction deficit D dominant direction ND non-dominant direction U18 under 18 U17 under 17 U16 under 16 U15 under 15

+ Significant (*p* < 0.0001) difference from ND by *t*-test analysis.

* Significant (*p* < 0.01) difference from U15 by one-way ANOVA analysis.

# Significant (*p* < 0.01) difference from U16 by one-way ANOVA analysis.

a Between-group difference over U17 with a *small* effect by robust statistical analysis.

b Between-group difference over U16 with a *moderate* effect by robust statistical analysis.

c Between-group difference over U16 with a *large* effect by robust statistical analysis.

d Between-group difference over U15 with a *small* effect by robust statistical analysis.

e Between-group difference over U15 with a *moderate* effect by robust statistical analysis.

Regarding robust statistical analysis, in U18 the AI% of COD_d_ was lower than the AI% in U17, U16, and U15 with a *small* (*d*_r_ = −0.39 [−1.09 to 0.30]), *large* (*d*_r_ = −0.97 [-1.07 to 0.23]), and *small* (*d*_r_ = −0.24 [-0.93 to 0.45]) effect, respectively (Table 2). In U17, the AI% of COD_d_ was also lower than AI% in U16 and U15 with a *moderate* (*d*_r_ = −0.67 [−1.38 to 0.04]) and *trivial* (*d*_r_ = −0.14 [−0.54 to 0.84]) effect, respectively (Table 2); likewise, in U16 the AI% of COD_d_ was higher than the AI% in U15 with a *moderate* effect (*d*_r_ = −0.75 [−147 to −0.03]) (Table 2).

**Figure 2:**
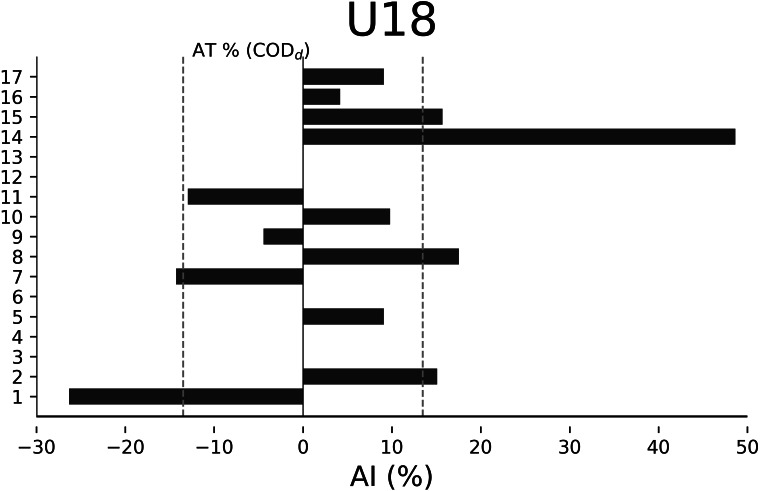
Individual asymmetry data (AI%) for change of direction total time (COD_t_) and deficit (COD_*d*_) within the U18 category. Bars oriented towards the right showed the asymmetry favoring the right limb, and bars oriented towards the left showed the asymmetry favoring the left limb. The dotted line indicates the asymmetric threshold of COD_d_.

**Figure 3:**
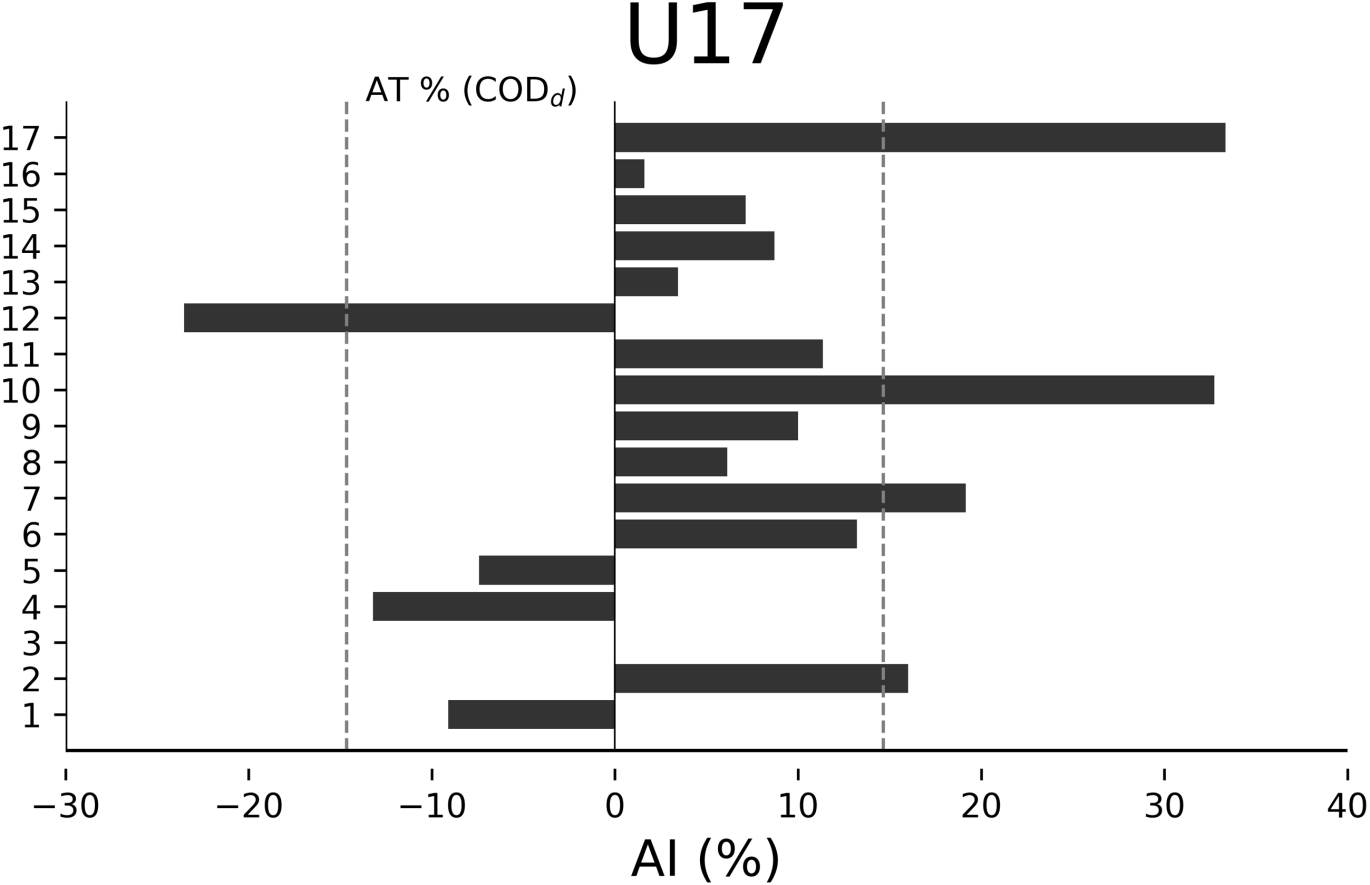
Individual asymmetry data (AI%) for change of direction deficit (COD_d_) within the U17 category. Bars oriented towards the right showed the asymmetry favoring the right limb, and bars oriented towards the left showed the asymmetry favoring the left limb. The dotted line indicates the asymmetric threshold COD_d_.

**Figure 4:**
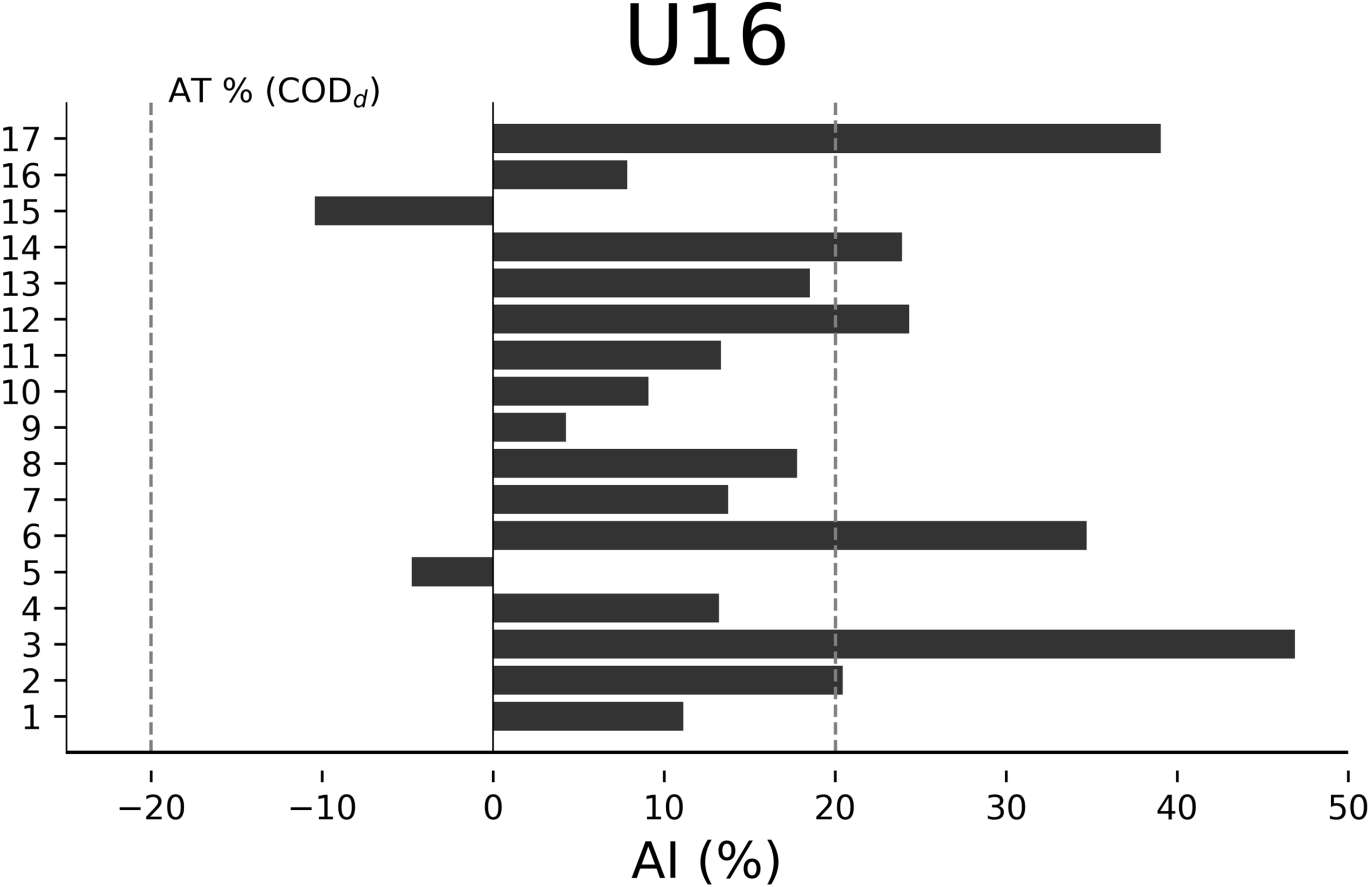
Individual asymmetry data (AI%) for change of direction deficit (COD_d_) within the U16 category. Bars oriented towards the right showed the asymmetry favoring the right limb, and bars oriented towards the left showed the asymmetry favoring the left limb. The dotted line indicates the asymmetric threshold of COD_d_.

**Figure 5:**
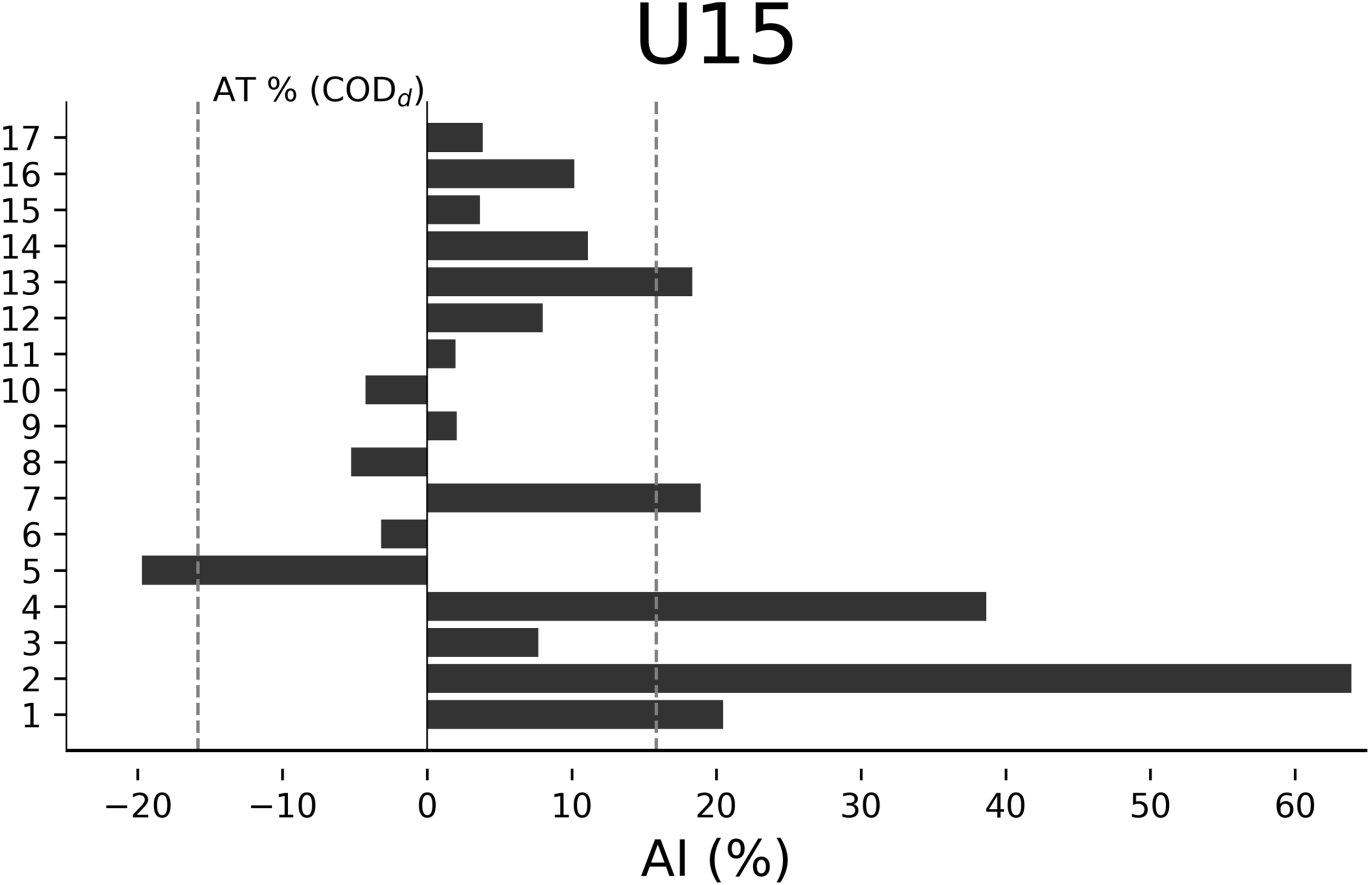
Individual asymmetry data (AI%) for change of direction deficit (COD_d_) within the U15 category. Bars oriented towards the right showed the asymmetry favoring the right limb, and bars oriented towards the left showed the asymmetry favoring the left limb. The dotted line indicates the asymmetric threshold of COD_d_.

The AT% of COD_d_ were −13.48% for U18 (Fig. 2), −14.65% for U17 (Fig. 3), −20.01% for U16 (Fig. 4), and −15.84% for U15 (Fig. 5), respectively. In U18, 6 out of 17 players were considered asymmetrical (Fig. 2), 5 out of 17 players were deemed asymmetrical in U17 (Fig. 3), 6 out of 17 players were considered asymmetrical in U16 (Fig. 4), and 6 out of 17 players were deemed asymmetrical in U15 (Fig. 5). Players exhibiting asymmetries greater than 10% threshold were 8 out of 17 (∼47%) in U18, 9 out of 17 (∼53%) in U17, 13 out of 18 (∼76%) in U16, and 8 out of 17 (∼47%) in U15.

## Discussion

The main findings of this study were: (i) asymmetries changed across age with the U16 exhibiting on average the highest AIs%; (ii) sprint performance improved linearly across age while U18 and U15 displayed fastest and slowest 10-m sprint and 90° COD total times (COD_t_), respectively. Moreover, when evaluated by COD deficit (COD_d_), 90°COD performance was similar across age categories.

To the best of the authors’ knowledge, this is the first study comparing asymmetries across age by COD_d_. It appears that AIs% tended to diminish in older players (≥16 years). This result seems to be in line with that previously reported by Atkins et al. (2016) in a sample of elite soccer players from U13 to U17. Although the authors examined bilateral differences in ground reaction forces during deep squat exercise, they showed substantial bilateral imbalance from U14 to U16 age groups, supporting that older players (≥ 16 years) are less prone to manifest asymmetry than their younger peers. The reason why asymmetry becomes notable in middle adolescence is still unclear. Changes in the body shape due to maturity-related variations might represent a possible explanation for the existence of bilateral imbalances. This could be attributed to potential changes in motor control that may arise during the growth spurt (Atkins et al., 2016). However, it has been previously demonstrated that players can exhibit substantial asymmetries despite displaying similar age at peak height velocity (Atkins et al., 2016). Accordingly, our age categories presented similar age at peak height velocity while displaying changes in AIs%, especially for U16. Nonetheless, caution should be applied when using this parameter, as it presents limitations. In this study, the age at peak height velocity was obtained by an indirect rather than direct assessment (e.g., bone density scan). Moreover, to obtain a clear picture of the individual’s maturity status, changes in body dimensions and inter-/intra-muscular coordination should be also considered in relation to both maturation and/or training. Of note, it has been observed that during maturation, young players may face a period of “motor awkwardness” (Rommers et al., 2019), which often worsens an individual’s ability to coordinate and control movements during complex tasks, such as COD. This condition may have impacted on the potential ability of U16 to change direction similarly in both sides. Unfortunately, very few studies addressed the topic of “motor awkwardness”, and longitudinal data are needed to provide a clear picture on the motor control behavior across age and adolescence spurt in terms of asymmetry assessment (Rommers et al., 2019). Other reasons may be sought toward the sporting specialism within adolescence. At this stage, sport-specific training sessions are aimed to specialize players’ mobility for a given role and movement direction on the pitch. This condition may be related to the need of soccer clubs to evaluate each individual player in the middle-to-late adolescence and decide for her or his career (Vaeyens et al., 2006). In this context, an accumulated sport-specific training exposure in specializing years (within U16s) may increase the directional asymmetry between the dominant and non-dominant side (Read et al., 2018). However, this seems to be in contrast with the lower AI% found in U17 and U18 groups, in which the exposure to sport-specific training is relevant. It can be speculated that the additional training strategies (e.g., strength training, injury prevention programs) experienced by the U17 and U18 would be a further neuromuscular stimulus (Atkins et al., 2016), possibly contributing in lowering directional asymmetries in these age groups. Nevertheless, given the paucity of information regarding asymmetry in youth, further studies are warranted to provide the literature with additional and extended knowledge on the association between asymmetry and training contents in youth and its potential change across age in soccer. In summary, the capability of COD_d_ to show asymmetries across age may be of practical relevance in the monitoring process of COD ability in young players.

The present findings are in line with the study of Dos’Santos et al. (2019b), which reported asymmetries of COD_d_ (in the 505 COD test) on average greater than 10% in youth netball players. Moreover, the authors found that asymmetries of COD_d_ were approximately fivefold greater than asymmetries of total time (−11.9% vs. −2.3%, respectively). It should be noted that such difference is primarily due the COD_d_ values themselves, which are smaller than total time values, resulting in AIs% based on deficit always substantially greater than AIs% based on total time (Dos’Santos et al., 2019b). This observation assumes practical relevance as substantially greater AI% values compared to smaller values may avoid misinterpretation of actual imbalances on COD ability. In the present study, the percentage of players exhibiting an AI% of COD_d_ ≥ 10% was 47–53% in U18, U17, and U15, and 76% in U16, with an overall number of 22 out of 68 players exceeding the AT%. Although asymmetries of total time were not considered, it can be assumed that they would have been substantially lower than COD_d_, possibly below the 10%, as previously shown (Dos’Santos et al., 2017). Indeed, an imbalance < 10% might be masked or underestimated if COD total time would be used. Conversely, higher-sized AIs% could induce coaching staff to better explore the extent of imbalance between the two directions to minimize their potential influence on COD performance (Dos’Santos et al., 2019b).

The U18 and U15 exhibited fastest and lowest performance in COD_t_, respectively, while their performance resulted similar in COD_d_. Moreover, Dos’Santos et al. (2019b) reported that COD_d_ and total time classified oppositely an individual’s COD ability. This result was also observed in young adult experienced cricketers during the same test (i.e., 505 test) (Nimphius et al., 2016). The reason why COD_d_ provides such an opposing or different COD profiling compared with total time may be largely ascribed to the fact that its evaluation is not affected by sprint performance. Indeed, solely using total time would approximately replicate sprint performance (Nimphius et al., 2016), as clearly evidenced by the current 10-m sprint values across age. The fact that no differences were observed in COD_d_ (in D and ND directions) across age could indicate that COD ability might be preserved with increasing age. Unfortunately, there is a dearth of research comparing COD ability along different age categories using COD_d_, thus comparative analyses are limited. To the author’s extent, only the study by Loturco et al. (2019) examined the evolution of COD_d_ across different age categories (i.e., U15, U17, U20, and Senior) in soccer players. Unlike the current results, the authors reported that COD deficit (calculated by means of average velocity values) underwent a gradual increase suggesting a progressive impairment of COD ability along age. One possible explanation could be a different approach in performing the COD test (20-m zig-zag test including 3 CODs at 100° angle). Another explanation could refer to the increases of soccer-specific training sessions within the specialization year, which could interfere with appropriate COD and sprint development (Loturco et al., 2019). Lastly, body mass increment related to growth might cause a decrease in COD and sprint performance. Noticeably, we found a progressive improvement in the current 10-m sprint performance across the age as also seen in the study by Loturco et al. (2019).

In summary, the current findings indicate that despite similar COD_d_ and progressive sprint performance improvements among the categories, COD directional asymmetry changed across age with the U16 exhibiting the highest imbalance. This assumes a practical relevance when dealing with adolescents experiencing a “motor awkwardness” phase. However, since scarce information exists on COD_d_ profile in youth soccer, further studies are needed to clarify the role of maturation, training content, and type of selected sprint and COD tests to detect asymmetry and COD ability.

This study presents three main limitations that should be acknowledged. The recruited players were sub-elite, thus, generalization on elite populations are limited. However, considering the study by Hart et al. (2014), it should be noted that the current age categories outperformed their elite peers on 10-m sprint and 90°COD tests. Additional studies involving elite soccer players with superior sprint and COD performance are needed to corroborate or not the present results on asymmetry across age. Another limitation is the lack of a general motor coordination assessment, as the information gained from COD_d_ across age alone does not provide conclusive evidence. This assessment would have informed on the presence of a potential growth-related movement impairment (motor awkwardness), thus providing additional and extended knowledge on the role of motor coordination in affecting directional asymmetry. A third limitation refers to the use of a specific technique (side-step maneuver) to change direction. Although we tried to control the technical execution rigorously, imposing a specific and equal movement pattern to all individuals might have limited the expression of the best performance for some of the players.

## Conclusions

This study demonstrated that asymmetries changed across age in youth soccer with U16 being, on average, the most asymmetrical age category. Moreover, sprint time improved linearly across age, while COD ability measured by COD_d_ did not differ across age. Given the results of this study, soccer strength and conditioning coaches are encouraged to assess asymmetries between dominant and non-dominant direction. Of note, even though COD ability might not change across age, its assessment is equally important for directional asymmetries and should be included to the field-based sport-specific testing tools in youth soccer.

##  Supplemental Information

10.7717/peerj.9486/supp-1 Supplemental Information 1 Raw data including both dominant (D) and non-dominant (ND) sides for 90° COD (named ”Cdd”) and deficit performance time Each row represents an individual data along with each dependent variables including also the asymmetric index. Data from the robust analysis are shown in two separate columns.

## Additional Information and Declarations

### Data Availability

The following information was supplied regarding data availability: The raw data of each age category are available in the Supplementary File.
